# Processing Optimization and Toxicological Evaluation of “Lead-Free” Piezoceramics: A KNN-Based Case Study

**DOI:** 10.3390/ma14154337

**Published:** 2021-08-03

**Authors:** Antonio Iacomini, Juan Antonio Tamayo-Ramos, Carlos Rumbo, Irem Urgen, Marzia Mureddu, Gabriele Mulas, Stefano Enzo, Sebastiano Garroni

**Affiliations:** 1 Department of Chemistry and Pharmacy, University of Sassari and INSTM, Via Vienna 2, 07100 Sassari, Italy; m.mureddu6@studenti.uniss.it (M.M.); mulas@uniss.it (G.M.); enzo@uniss.it (S.E.); sgarroni@uniss.it (S.G.); 2 International Research Centre in Critical Raw Materials—ICCRAM, Universidad de Burgos, Plaza Misael Bañuelos s/n, 09001 Burgos, Spain; jatramos@ubu.es (J.A.T.-R.); crumbo@ubu.es (C.R.); iremurgen93@gmail.com (I.U.); 3 Department of Metallurgical and Materials Engineering, Meselik Campus, Eskisehir Osmangazi University, 26480 Eskisehir, Turkey

**Keywords:** lead-free piezoceramics, sodium potassium niobate, processing, toxicity assays, X-ray diffraction, mechanochemistry

## Abstract

Due to the ever-increasing limitations of the use of lead-based materials, the manufacturing of lead-free piezoceramics with competitive piezoelectric properties and established nontoxicity is considered a priority for the scientific and industrial community. In this work, a lead-free system based on sodium potassium niobate (KNN), opportunely modified with MgNb_2_O_6_ (MN), was prepared through a combination of a mechanochemical activation method and air sintering, and its toxicity was evaluated. The effect of the mechanical processing on the microstructure refinement of the processed powders was established by X-ray diffraction and the average crystallite size content of the Nb_2_O_5_ species was evaluated. The experimental evidence was rationalized using a phenomenological model which permitted us to obtain the amount of powder processed at each collision and to optimize the activation step of the pre-calcined reagents. This influenced the final density and piezoresponse of the as-sintered pellets, which showed optimal properties compared with other KNN systems. Their toxicological potential was evaluated through exposure experiments to the pulverized KNN-based pellets, employing two widely used human and environmental cellular models. The in vitro assays proved, under the selected conditions, the absence of cytotoxicity of KNN-bases systems here studied.

## 1. Introduction

Nowadays, piezoceramics represent an undeniable class of functional materials that are largely exploited in a wide range of key technologies, including imaging probes for medical diagnostics, new generations of sonars and wireless admittance monitoring systems [1]. With exceptional electromechanical properties and mild processing conditions, highly dense PZT (lead, zirconate, titanate), with a general formula Pb(Zr_x_Ti_1−x_)O_3_, is the most commonly used compound in the manufacturing of piezoceramics-based devices [2,3,4]. However, despite the excellent properties exhibited, the use of highly toxic lead oxide as starting reagent has led the industrial and scientific community to address their efforts in the development of competitive alternatives composed of lead-free materials [5]. Among the many lead-free based candidates, KNN (sodium, potassium, niobate), with a general formula of K_x_Na_1−x_NbO_3_, has received great interest in the recent past, due to its high d_33_ value (390–400 pC/N) coupled with a high Curie temperature (217–304 °C) [6,7,8,9,10]. These promising properties, combined with the ever-increasing restrictions adopted against the use of lead in the manufacturing of costumer objects, have promoted fervent research into these systems [11]. However, their extensive penetration into the commercialized devices is still hampered by their scarce reproducibility, poor sinterability and as yet unclarified impact on human health.

The processing of the KNN-based systems results is affected by many open issues. Firstly, alkali loss during the calcination and sintering steps, usually performed at high temperatures (700 °C and 1150 °C, respectively), contributes to an incorrect stoichiometry with the further formation of undesired secondary phases which drastically reduce the performance of the system and its reproducibility [12]. Likewise, KNN-based powders with excessively large particles and grain sizes result in it being difficult to sinter under mild conditions. These processes-related problems can be overcome by increasing the reactivity of the pre-calcined powders by mechanical processing or introducing specific dopants [13]. Recently, Galassi and coauthors, activating the pre- and post- calcined powders by prolonged high-energy ball milling, obtained high dense pellets of KNN (>94%), characterized by good piezoelectric properties (d_33_ = 97 pC/N) and a mild calcination temperature of 700 °C [14]. In addition, the introduction of additives, such as copper and magnesium-based oxides, significantly improved the densification of the KNN pellet, which is ascribable to the formation of a liquid phase during the sintering step [15].

Concerning the toxicological aspect of KNN-based materials, few tangible results have been published to the best of our knowledge. There are few works focusing on the environmental impact and sustainability of KNN, which is expected to be less hazardous than PZT, but the mining of the starting reagents (Nb_2_O_5_) has been evaluated as being more environmentally deleterious if compared with lead oxide [16,17,18]. A toxicological experimental assessment and information about the safety of KNN materials are then of primary importance, together with their processing optimization.

In the light of the above-mentioned state of the art, this work has been focused on the optimization of the processability of KNN, which was successfully accomplished by exposing the pre-calcined starting reagents to high-energy ball milling. Three batches of K_0.5_Na_0.5_NbO_3_, a pure KNN and two modified KNNs with increasing amounts of MgNb_2_O_6_ (MN) ternary oxide, have been densified and the piezoresponse tested. With the aim to shed further light on possible hazard of KNN-based ceramics, the toxicological potential of the as-procced KNN pellets and commercial PZT were determined by in vitro screening employing two model organisms that were selected as representatives of human (A549 cell line) and environmental exposures (*Saccharomyces cerevisiae*).

## 2. Materials and Methods

### 2.1. Ceramic Preparation

KNN powders were prepared through solid-state reaction, starting from a mixture of K_2_CO_3_ (Sigma Aldrich, St. Louis, MO, USA, ≥99.995%), Na_2_CO_3_ (Sigma Aldrich, St. Louis, MO, USA, ≥99.5%) and Nb_2_O_5_ (Alfa Aesar, 99.9985%), in the stoichiometric molar ratio 1:1:2, respectively. Manipulations of the starting reagents were conducted in an Ar Glove box machine (MBraun) to prevent hydration, contamination and side reactions. We transferred 8 g of powders into a stainless-steel vial together with 1 ball (stainless steel) of 10 g. The powders were mechanically treated for 24 h, at 875 rpm, by using a Spex 8000M Mixer/Mill. The calcination step was conducted from room temperature to 825 °C for 4 h using a heating rate of 3 °C/min and then cooled down to 25 °C with a cooling rate of 10 °C/min.

MgNb_2_O_6_ powders were synthesized through solid-state reaction. MgO (Sigma Aldrich, St. Louis, MO, USA, ≥99%) and Nb_2_O_5_ (Alfa Aesar, 99.9985%) were mixed by high energy ball-milling, in a stoichiometric molar ratio 1:2, (Spex 8000M Mixer/Mill), at different milling times, and then thermally treated up to 1000 °C (dwell time: 1 h) by using a heating rate of 5 °C min^−1^ (the cooling step was realized with a rate of 10 °C min^−1^). The final product was characterized by X-ray diffraction (see Figure S1 in the Supplementary Materials). The modification process of KNN powders was made by mixing appropriate amounts of KNN and MN: three samples were prepared with increasing weight percentage of MN (0, 0.5, 1 wt.%, respectively). KNN and MN were milled with 5 mL of ethanol (Sigma Aldrich, St. Louis, MO, USA, purity > 95%), for 24 h. The as-obtained slurry was then transferred in a beaker and heated in an oven at 150 °C for 4 h to eliminate the solvent. The powders were finely ground in a mortar to obtain a fine particulate matter and mixed with few drops of polyvinyl alcohol (PVA) solution (3 wt.%) before compacting into a disk by means of a hydraulic press (220 kg/cm^2^ for 30 min). The pellets were thermally treated for 10 h at 550 °C to eliminate all traces of PVA. Sintering was conducted at 1100 °C for 3 h using a heating rate of 5 °C/min and cooling rate of 10 °C/min. Finally, bulk densities were measured by geometric method.

### 2.2. Materials Characterization

Structural investigations were conducted using a SMARTLAB diffractometer with a rotating anode source of copper (𝜆Cu Kα = 1.54178 Å) working at 40 kV and 100 mA. The spectrometer is equipped with a graphite monochromator and a scintillation tube in the diffracted beam. Quantitative analysis of the crystalline phases and structure determinations were performed with the MAUD software (Materials Analysis Using Diffraction), a Rietveld extended program [19]. Lattice parameters of the constituent phases were refined from the line peak positions after allowing a correction for the zero-offset, while crystallite size and lattice disorder contributions to the peak broadening were separated because of the wide angular range explored. Microstructure and morphology of the samples were characterized by Quanta FEI 200 scanning electron microscope (SEM). Thermogravimetric were performed with Labsys TGA/DTA and DSC instrument from room temperature to 1000 °C using a heating rate of 5 °C/min and cooling rate of 30 °C/min under argon atmosphere. Disc-shaped samples with thicknesses 1 mm and with 13 mm diameter were used for electrical property characterization. The direct piezoelectric coefficient (d_33_) was measured by using a Berlincourt d_33_ meter (SINOCERA YE2730Q) at 100 Hz. Before measuring the pellets were exposed to an electric field at 130 °C to initially poled samples with a maximum of 35 kV/cm.

### 2.3. Toxicology Assessment

#### 2.3.1. Human Alveolar A549 Cells Viability Assay

The cell line A549 (ATCC, CCL-185) was grown and maintained in a humidified incubator (37 °C and 5% CO_2_) with DMEM, supplemented with 10% FCS and 1% penicillin/streptomycin (Sigma-Aldrich). To assess the potential toxicity of KNN powders, human lung carcinoma A549 cells were exposed to different concentrations, for a period of 24 h. First, cells (approximately 3 × 10^4^) were incubated in culture media with 5% CO_2_, for 24 h at 37 °C. Subsequently, they were seeded in 96-well plates, and exposed to the KNN powders, diluted in DMEM 1% FCS. Once the cells were exposed for 24 h, they were washed and incubated for 2.5 h, with 100 μL of a Neutral Red solution, prepared as follows: a 1:100 dilution (in treatment media) from the Neutral Red stock (4 mg/mL) was incubated for 24 h at 37 °C, protected from light. Afterwards, cells were washed with DPBS and fixed with formaldehyde 4%. Cells were washed again with DPBS, and an extraction solution, containing 50% ethanol 96°, 49% distilled H_2_O and 1% acetic acid, was added to each well. After a moderate shaking step of 10 min, the extraction solution was transferred to an opaque 96-well plate, and fluorescence was measured employing a microplate reader (BioTek Synergy HT, excitation: 530/25; emission: 645/40). Results were expressed as percentage of the average values obtained in the negative control condition.

#### 2.3.2. *Saccharomyces cerevisiae* Viability Assay

The *S. cerevisiae* BY4741 strain was grown and maintained in standard liquid YPD medium (1% yeast extract, 1% yeast bacto-peptone, 2% glucose). Cell cultures in liquid media were placed on a rotary shaker at 185 rpm at 30 °C. Yeast cells viability after exposure to different KNN concentrations was determined by performing the colony forming units (CFUs) assay, through the following steps: Yeast cells at OD600 = 1 (exponential growth phase) were exposed to 1000 μg/mL of KNN and KNN-based powders for 2 and 24 h, in 1 mL cultures, using 24-well plates. To determine CFUs at both sampling times, 100 μL of cells aliquots, previously diluted 10^4^ times, in the case of 2 h exposure, and 10^5^ times, in the case of 24 h exposure, were spread on solid YPD medium (6% agar), employing disposable Digralsky spatula. Subsequently, agar plates were incubated at 30 °C for 48 h. Afterwards, colony forming units were counted for each condition tested.

## 3. Results and Discussion

### 3.1. Ball Milling Effect on Mixed Powders

**Figure 1:**
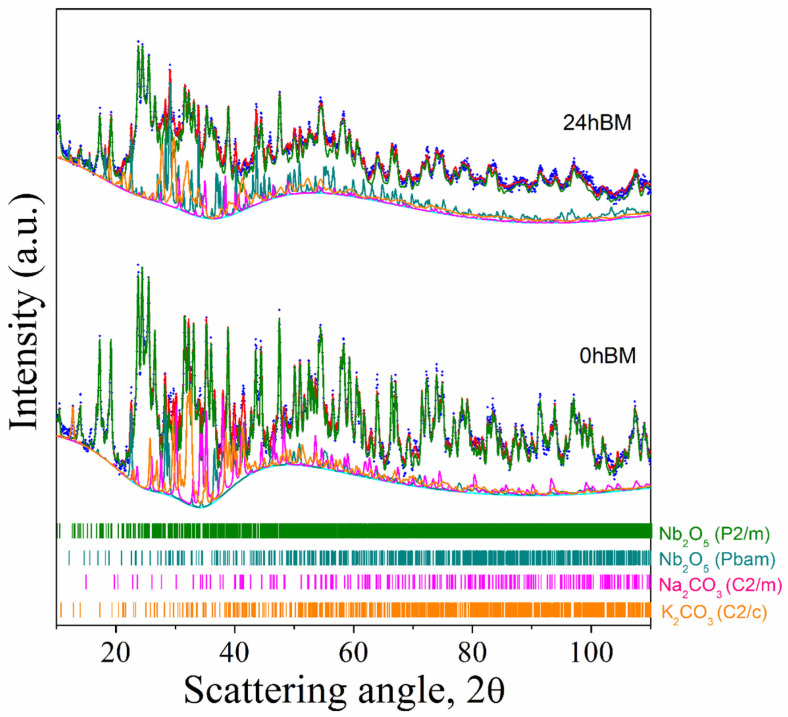
XRD pattern of the reaction mixture before (0 h BM) and after (24 h BM) the mechanical treatment. Blue dots are experimental data points, cyan lines are the background, red lines are the full calculated pattern and colored lines represent the contribution of each phase to the theoretical model used for the calculation.

Figure 1 shows the analysis investigation referring to the as-mixed powders prior to any mechanical treatment (0 h BM). The integral agreement factor calculated from the residual curve amounts to Rwp = 14.88%, which is assumed satisfactory, given the complex phase composition in the pattern and the narrow peaks, mainly from the Nb_2_O_5_ monoclinic phase, dominating the pattern.

In this fit, the instrument function, determined separately with a well-crystallized LaB6 specimen, is considered. The specimen appears to be composed of both monoclinic (68 wt.%) and orthorhombic (1 wt.%) polymorphs of Nb_2_O_5_, with net prevalence of the first. The numerical processing is also able to assess the presence of Na_2_CO_3_ (15 wt.%), despite its lower electron density with respect to niobia. The graph also shows (orange curve) the presence of hydrated potassium carbonate (16 wt.%) (the X-ray data collection was conducted under air). The average crystallite size of monoclinic niobia, after correction for the instrument function, is ca 1050 Å.

Concerning the 24 h BM sample, from rapid spectrum analysis it is possible to deduce that no substantial new phase was formed during mechanical grinding. Moreover, a broadening of the peaks was observed. This broadening could be caused by microstrain introduced by mechanical stress during the milling and/or by fragmentation and decrease in the average crystallite size down to the nanometer range [20]. The average crystallite size for monoclinic niobia is 400 Å, and the amount of the orthorhombic polymorph of niobia is increased in this specimen (about 11% wt., to compare with 1% wt. in the 0 h BM min specimen). The increase in the orthorhombic polymorph after the milling step is consistent with previous work [21]. The total amount of the niobia monoclinic polymorph is roughly the same (71 wt.%); this is mainly due to the lower detectability of sodium and potassium carbonates after the milling process, which were detected with a percentage by weight, respectively, of 8 and 10%. The perovskite phase of KNN was not detected in this sample, or at least, its concentration is below the detection limit of the instrument.

**Figure 2:**
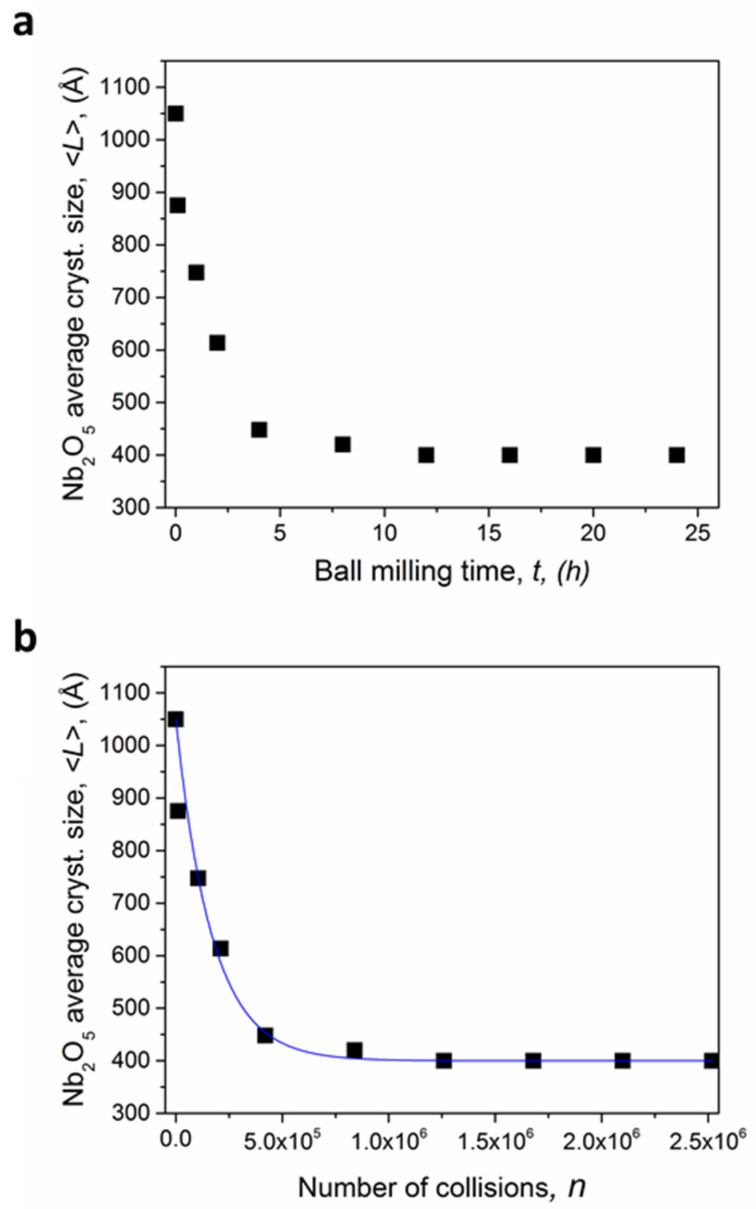
Average crystallite size <L> of coherent diffraction domains as a function of the milling time t_m_ (**a**) and total number *n* of collisions (**b**).

Considering the direct relation between crystallite sizes and powders’ reactivity, the refinement of the former represents an important aspect that needs to be properly investigated. To this regard, the evolution of the microstructure of the starting powders submitted to BM was then evaluated from the sequence of XRD patterns, not shown here for brevity, acquired for selected times up to 24 h of mechanical processing. The values of the coherent diffraction domains are then shown in Figure 2 as a function of the milling time (Figure 2a). The increasing milling time was accompanied by the progressive decrease in crystallite size <L>. The monotonic change reached an asymptotic value after about 11 h of milling: the average crystallite size <L> varied from an initial value L_0_ of about 1050 Å (0 h BM) to a final one L_f_ of 400 Å (12–24 h BM).

Taking in consideration the experimental approach adopted for this work (8 g of powders, 1 ball, 875 rpm), an inelastic regime of collisions was assumed during the mechanical process, which should provide a regular milling dynamic. Under such conditions, the milling time, t_m_, and the number of impacts, *n*, show a direct proportionality, as also indicated in [22], and the variation of the average crystallite size can be also reported as a function of the total number of collisions (Figure 2b). It is in fact well established that mechanochemical processing occurs during each collision and the powders involved in this event are only a subvolume V* of the trapped powder. The stochastic model previously elaborated and discussed in detail in [22], can be applied to the variation of physical properties, such as the refinement of crystallite size during a series of impacts. The kinetic of microstructural refinement can be described by the Equation (1):

(1) $$\langle L\rangle =L_{0}\exp\left(-Kn\right)+L_{f}\left[1-\exp\left(-Kn\right)\right]$$

where *n*, *L*_0_ and *L_f_* have been previously introduced, while *K* is the apparent rate constant of the process which corresponds to the volume fraction effectively processed at collision (K = V*/V_tot_). The mathematical function Equation (1) describes almost perfectly the experimental values reported in Figure 2b (full blue line and black squares, respectively). The fit provides a *K* value of 5.92 × 10^−6^, which indicates that a mass of 34.6 μg is processed per collision. According to the model used it is then possible to rationalize that the energy transferred to the trapped powders at first collision is enough to provide the change of the initial *L_0_* grain size to the final *L_f_* grain size in the 34.6 μg of mass processed. Further collisions do not cause any noticeable change. Furthermore, it is possible to establish that operating with an impact energy, *E_imp_*, of 0.088 J, the reduction of Nb_2_O_5_ crystallize size is accomplished after 1.20 × 10^6^ collisions (≈12 h BM). This allowed us to optimize this processing step, significantly reducing the milling time and preventing any contaminations induced by prolonged ball milling. These milling parameters were then adopted for producing the KNN-based powders for being densified and then tested. Further characterization on the as processed powders was performed to confirm the influence of the milling processing.

**Figure 3:**
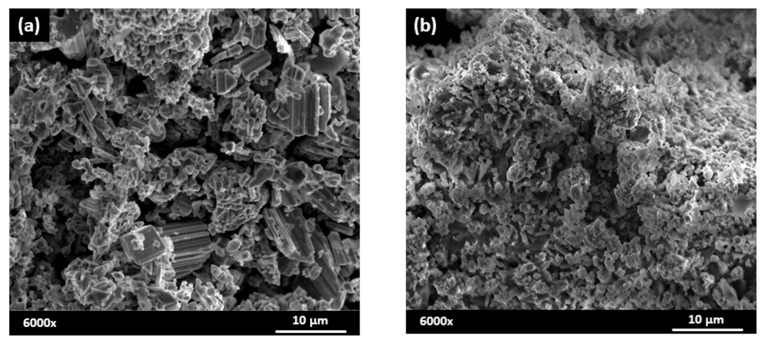
SEM micrograph of mixture powders: (**a**) 0 h BM and 12 h BM (**b**).

Representative micrographs of sample, a mixture of K_2_CO_3_, Na_2_CO_3_ and Nb_2_O_5_ prepared before and after mechanical treatment of 12 h, are reported in Figure 3. In Figure 3a the sample appears only as a simple mixture of Nb_2_O_5_ and carbonates. In Figure 3b these structures have been reduced in size by the mechanical grinding and the mixture appears as an aggregate of fine particulate matter. The mechanical treatment induces important morphological and structural changes to the reaction mixture, and this may affect the solid-state reaction.

**Figure 4:**
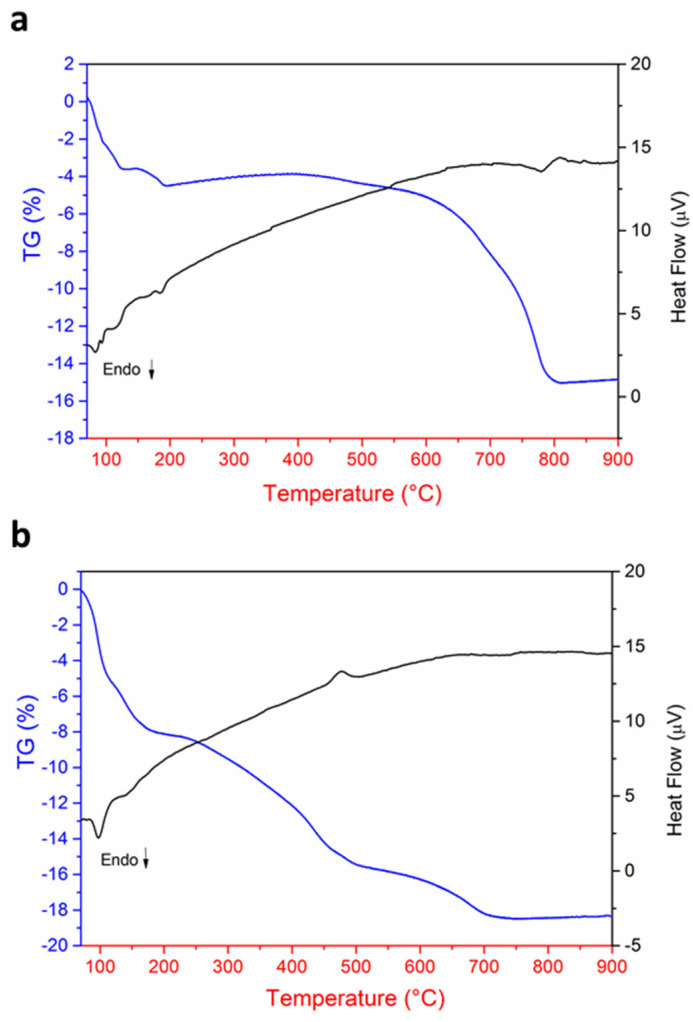
Thermogravimetric analyses and DSC of mixed powders mechanically treated for (**a**) 0 h BM and (**b**) 12 h BM. The blue line is the thermogram and the black line is the DSC signal.

Thermogravimetric and DSC signals have been acquired on the samples under consideration to further investigate any reactivity differences of the powder mixture (Figure 4). All the experiments were carried out from room temperature up to 1000 °C. It can be seen from Figure 4a the powder mixtures of the unmilled specimen go through two stages of thermal decomposition from room temperature to about 125 °C, which are accompanied by two small endothermic peaks. According to Farooq et al., the first weight loss is due to the evaporation of physically adsorbed water, while the second may be due to the removal of chemically adsorbed water [23]. Between 150 °C and 190 °C there is another weight loss of~1 wt.%, which could be related to the decomposition of some residuals of the hydrogen carbonates [24]. Between 470 and 800 °C it can be seen that a large weight loss of about 10.5% occurs, which is due to the decomposition of the alkali carbonates. The thermogravimetric profile of the milled sample (Figure 4b) is quite different from the previous one From room temperature to~170 °C it is possible to see two clear weight losses of about 7.8 wt.% accompanied by two endothermic peaks correlated to the removal of water. After that, two distinct thermal phenomena take place, between 240–500 °C and 600–700 °C, respectively, which are responsible for a relative weight loss of 10.7 wt.% due to the decomposition of the alkali carbonates. Considering the reaction that takes place between the carbonates and niobium oxide, it is possible to calculate the theoretical weight loss which occurs during the heating treatment:

(2) $$K_{2}{\mathrm{CO}}_{3}+{\mathrm{Na}}_{2}{\mathrm{CO}}_{3}+2{\mathrm{Nb}}_{2}O_{5}\to 4(K_{0.5}{\mathrm{Na}}_{0.5}){\mathrm{NbO}}_{3}+2{\mathrm{CO}}_{2}↑\left(g\right)$$

From Equation (2) it can be deduced that the weight loss is due to the formation of two moles of carbon oxide. Therefore, the estimated weight loss is 88.018 g/mol (CO_2_ M.W = 44.009 g/mol), which corresponds to about 11.3 wt.%. In the experimental case, the contribution of the weight loss of the adsorbed water must also be considered. In both samples, the weight loss due to the decomposition of the carbonates is in good agreement with the theoretical calculation. The different reactivity is well highlighted by the different trends of the two thermograms. Indeed, the decomposition of the carbonates, in the case of the milled sample, begins relatively early at around 240 °C and is completed at 700 °C, while for the unmilled sample this range is between 470–800 °C. Therefore, the milling process considerably increases the reactivity and lowers the calcination temperature of the reaction.

**Figure 5:**
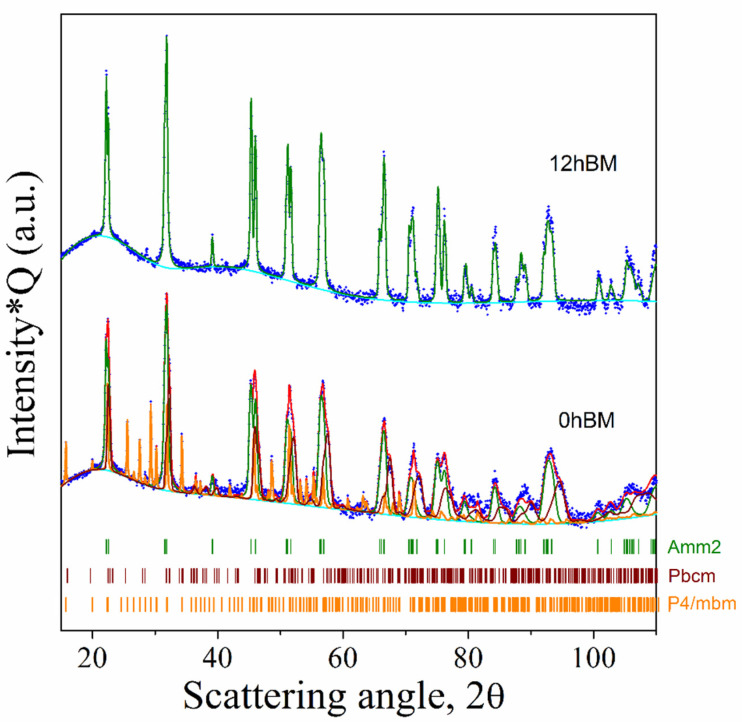
XRD patterns of KNN powders collected from thermogravimetric experiments. The logarithmic intensity is multiplied times Q (2π/d_hkl_) to emphasize the high angle peaks.

The powders from thermogravimetric analyses were preserved and analyzed by XRD. The results are shown in Figure 5. The pattern of the 0 h BM sample can be decomposed, according to Rietveld, as it is made by three phases: orthorhombic potassium niobate (s.g. *Amm2*), orthorhombic sodium niobate (s.g. *Pbcm*) and tetragonal K_6_Nb_10.8_O_30_, with a weight percentage about of 66%, 17% and 17%, respectively. The low matching value reached (R_wp_ = 5.51%) highlights the excellent approach of the model to the experimental data. The tetragonal P4/mbm phase has the tungsten–bronze type structure with the formula K_6_Nb_10.8_O_30_. This phase is formed in case of alkaline ions deficiency, and it is an intermediate phase during the solid-state reaction [25]. It is also frequently reported in KNN sintered samples as it is formed during the sintering step due to the volatilization of the alkaline ions [26].

The orthorhombic *Pbcm* is an antiferroelectric phase with formula NaNbO_3_, while the orthorhombic *Amm2* phase is the well-known ferroelectric phase commonly reported for pure KNN systems [9]. Otherwise, the 12 h BM sample shows a single-phase structure with orthorhombic symmetry (s.g *Amm2*). A small peak has been detected around 28° which is most probably related to some residue of the tetragonal tungsten bronze phase. The cell parameters (a = 3.9444 Å; b = 5.6408 Å; c = 5.6723 Å) and volume of the cell (V = 126.206 Å^3^) are in good agreement with those reported for pure stoichiometric K_0.5_Na_0.5_NbO_3_ (a = 3.9436 Å; b = 5.6510 Å; c = 5.6726 Å; V = 126.415 Å^3^) [8]. The lack of homogenization and low reactivity of the starting powders determine the presence of a niobate mixture, even after a high temperature heat treatment (1000 °C). The milling step increases the reactivity, lowers the calcination temperature and allows us to obtain a single-phase powder product.

### 3.2. Sintered KNN-xMN Ceramics and Piezoelectric Properties Assessment

**Figure 6:**
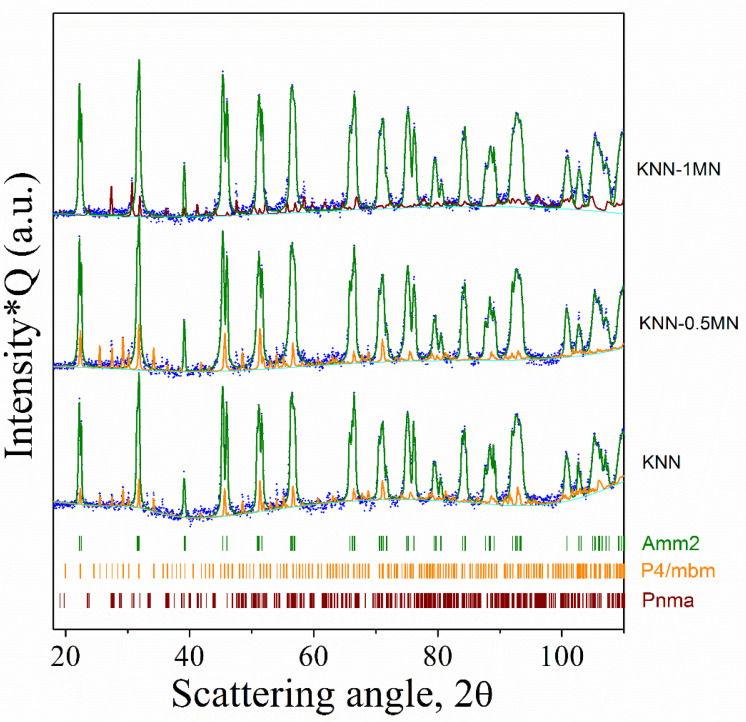
XRD patterns of KNN-xMN sintered pellets.

MN was then added to calcined KNN powders in different amounts (0, 0.5 and 1 wt.%) and sintered at 1100 °C. The XRD patterns of the sintered pellets are shown in Figure 6. All the three samples show a predominance of the perovskite phase with orthorhombic symmetry and *Amm2* space group. For the KNN and KNN-0.5MN samples, the trace presence (<3 wt.%) of the tetragonal P4/mbm phase was observed, most likely due to the volatilization of alkaline ions during the sintering process. The KNN-1MN sample shows a different impurity phase with orthorhombic symmetry and Pnma space group.

**Figure 7:**
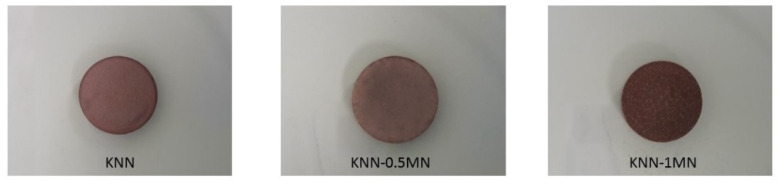
Pictures of KNN-xMN sintered pellets.

This phase possesses a KTiNbO_5_-type structure, also observed in other ceramics such as Rb(Mg_0.34_Nb_1.66_)O_5_ and K(Fe_0.43_Nb_1.57_)O_5_; however, its composition is to be clarified and required further investigations. Figure 7 shows the images of the sintered pellets. All the samples have a brownish color which becomes even darker for high amounts of MN (1 wt.%). The addition of MN slightly increases the density of KNN (4.02 g/cm^3^) from 4.10 g/cm^3^ of KNN-0.5MN to 4.28 g/cm^3^ of KNN-1MN. These values correspond to 89%, 91% and 95% with respect to their theoretical densities (KNN = 4.510 g/cm^3^; KNN-0.5MN = 4.512 g/cm^3^; KNN-1MN = 4.514 g/cm^3^)

The piezoresponse of the as-poled densified materials have been tested and in Table 1 the main electrical properties of KNN-xMN ceramics, including a comparison with some relevant KNN prepared through mechanochemical activation, are summarized.

As it can be seen, the preliminary electrical properties of the KNN-xMN samples, prepared from the mechanically activated pre-calcined powders, are comparable with the references inserted in Table 1. Moreover, the addition of MN significantly increases the piezoresponse, probably ascribable to an increase in the density of the as-doped cylinders. It should be mentioned that the pellets prepared starting from the pre-calcined powders milled for 0 and 2 h (which corresponds to Nb_2_O_5_ crystallite sizes of 1050 Å and 620 Å, respectively), were characterized by the presence of secondary phases which decreased significantly the piezoresponse of the final pellets (44 and 56 pC/N, respectively). This result suggests that the refinement of microstructure induced by mechanical processing, in the pre-calcined powders, is a crucial parameter for manufacturing KNN pellets with a good piezoresponse [27]. However, despite the progress made in this direction, further efforts must address the characterization of the electromechanical properties and the temperature dependence of the dielectric permittivity of the pellets.

### 3.3. Toxicology Assessment Using Adenocarcinoma A549 Human Cells

**Figure 8:**
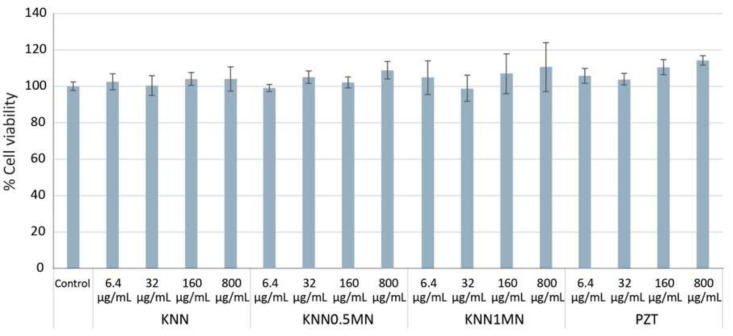
A549 cells viability after exposure to different concentrations of KNN, KNN-0.5MN, KNN-1MN, and PZT. “Control” corresponds to non-exposed cells.

To assess the potential cytotoxic effects of the different piezoelectric ceramics under study, the human lung carcinoma cell line (A549) was selected as a cellular model, to study possible adverse effects in human health via inhalation exposure of the selected piezoceramic powders. To determine the percentage of living cells after the exposure to different lead-free KNN systems (KNN, KNN-0.5MN and KNN-1MN) and to a commercial system (PZT, 3N-99.9%, provided by American Elements), the Neutral Red assay (one of the viability tests most widely used in nanotoxicological studies) was performed. A549 cells were exposed to different concentrations (6.4, 32, 160, 800 μg/mL) of the different samples for a period of 24 h. As shown in Figure 8, after A549 cells exposure to KNN, KNN-0.5MN, KNN-1MN, and commercial PZT, the viability of the cells was not reduced in the presence of any of the concentrations tested, indicating the absence of cytotoxicity in the employed conditions. Previous studies have reported the good biocompatibility of PZT piezoelectric ceramics, despite the risk of PB release into the environment after their preparation and disposal [28]. Additionally, the cytotoxicity of KNN has been reported to be low [18]. In fact, the biocompatibility of KNN ceramics has also been verified by employing MC3T3-E1 osteoblasts, being suggested as an appropriate biomaterial with electroactivity for bone tissue regeneration [29]. Therefore, the obtained results in the present work are in line with those previously reported in similar studies.

### 3.4. Toxicology Assessment Using the Model Yeast Saccharomyces Cerevisiae

**Figure 9:**
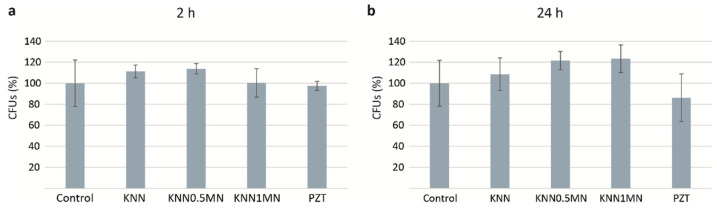
Determination of *S. cerevisiae* cells CFUs after exposure to 1000 μg/mL of KNN, KNN-0.5MN, KNN-1MN, and PZT, for 2 h (**a**) and 24 h (**b**). “Control” corresponds to non-exposed cells.

The yeast *S. cerevisiae* is a eukaryotic model that is extensively used to comprehend fundamental molecular mechanisms and biological processes, which is also used as a tool for the toxicological evaluation of substances. Therefore, to evaluate the potential environmental impact of BN, yeast cells were exposed to 1000 μg/mL of KNN, KNN-0.5MN, KNN-1MN, and PZT, at two exposure times (2 and 24 h), and subsequently their viability was assessed through colony forming units’ (CFUs) determination. As shown in Figure 9, no significant cells’ viability changes were observed after 24 h, amongst different exposure conditions. The antimicrobial properties of KNN ceramics are scarce, although they have been previously explored employing nosocomial bacteria [30]. However, data on their antifungal potential are not available. In contrast to the reported significant antibacterial effect of KNN piezoceramics against *Staphylococcus aureus*, the present study indicates that the materials under study do not have antifungal capacity. In any case, more studies are necessary to clarify the potential toxicity of KNN-based materials against different fungal species.

## 4. Conclusions

This work provides new experimental insights into the manufacturing and potential toxicity of the lead-free sodium potassium niobate (KNN) piezoceramics. Summarizing, KNN and KNN-MN ceramics were successfully synthesized through the mechanical activation of the starting reagents conducted with a shaker ball mill (Spex) under a regime of inelastic collisions and energy impact of 0.088 J. It was shown that the methodological approach adopted allows us to rationalize the microstructural refinement processes in the Nb_2_O_5_ species. The phenomenological model was able to describe the kinetics of crystallite size variation and the value of the kinetic constant indicates that 34.6 μg of powder are processed in a single collision. It was also established that only one collision event was enough to refine the crystallite size of the fraction of powder processed. Controlling these parameters makes possible the synthesis with a proper stoichiometry of KNN and significant reproducibility; at the same time, they could be useful for an upscaling of the process. Furthermore, it was possible to obtain pellets of KNN and KNN-xMN, which possessed high physical density (4.28 g/cm^3^) and good piezoelectric properties (97 pC/N), in particular for the MN modified systems. Finally, the toxicological test performed by exposing all the systems to two human and environmental cellular models, suggests that KNN-based materials are not cytotoxic and, in contrast with previous studies, that they do not have antifungal capacity.

## Figures and Tables

**Table 1:** Room temperature piezoelectric properties of KNN-xMN ceramics and a comparison with those of KNN reported in the current literature and prepared through mechanochemical activation route.

Property\Sample	KNN	KNN-0.5MN	KNN-1MN	KNN	KNN	KNN
*d*_33_ (pC/N)	78	97	92	97	95	78
ρ (% TD)	89	91	95	91	/	96
Ref.	[This work]	[This work]	[This work]	[14]	[20]	[26]

## Data Availability

Data are contained within the article.

## Supplementary Materials

The following are available online at https://www.mdpi.com/article/10.3390/ma14154337/s1, Figure S1: XRD pattern and Rietveld analysis of MgNb_2_O_6_.
