# Supplementary material for: Processing Optimization and Toxicological Evaluation of “Lead-Free” Piezoceramics: A KNN-Based Case Study
Source: Materials (Basel). 2021 Aug 3;14(15):4337. doi: 10.3390/ma14154337 (PMC8348597; doi:10.3390/ma14154337)
Supplement: Supplementary file 1 [file materials-14-04337-s001.zip › materials-1301595-supplementary.pdf]

Supplementary materials

Processing optimization and toxicological evaluation of "lead-free" piezoceramics: a KNN-based case study

Antonio Iacomini[1*], Juan A. Tamayo Ramos[2], Carlos Rumbo[2], Irem Urgen[2,3], Marzia Mureddu[1], Gabriele Mulas[1], Stefano Enzo[1], Sebastiano Garroni[1*]

[1]Department of Chemistry and Pharmacy, University of Sassari and INSTM, Via Vienna 2, I-07100 Sassari, Italy; aiacomini@uniss.it, m.mureddu6@studenti.uniss.it, mulas@uniss.it, enzo@uniss.it, sgarro-ni@uniss.it

[2]International Research Centre in Critical Raw Materials-ICCRAM, Universidad de Burgos, Plaza Misael Bañuelos S/n, 09001, Burgos, Spain; crumbo@ubu.es, jatramos@ubu.es, iremurgen93@gmail.com

[3] Department of Metallurgical and Materials Engineering, Eskisehir Osmangazi University, Meselik Campus 26480, Eskisehir, Turkey.

* Correspondence: aiacomini@uniss.it; sgarroni@uniss.it; Tel.: +39 079 229558

1. Synthesis of MN

The X-ray pattern of $MgNb_2O_6$ (MN) synthesized by solid-state method is shown in **Figure S1**.

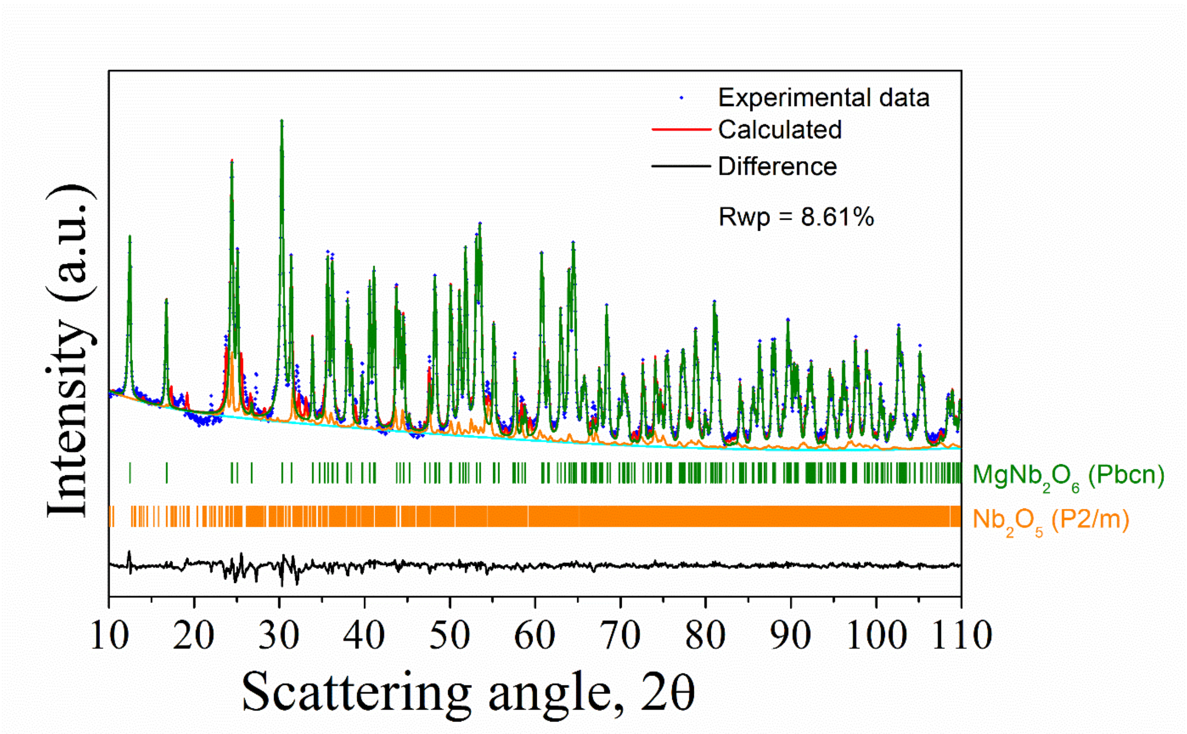

Figure S1. XRD pattern and Rietveld analysis of $MgNb_2O_6$.

A columbite-like phase $MgNb_2O_6$ (MN) with orthorhombic structure (s.g. Pbcn) was obtained; traces of unreacted niobium pentoxide (below 4%) was also detected.
